# Pregnancy and perinatal outcomes following exposure to GLP-1 and dual GLP-1/GIP receptor agonists: a time-window-stratified systematic review and meta-analysis

**DOI:** 10.3389/fphar.2026.1994366

**Published:** 2026-10-16

**Authors:** Erkan Mavigök, Ayşe Nur Mavigök

**Affiliations:** 1 Department of Obstetrics and Gynecology, MegaPoint Hospital, Kahramanmaraş, Türkiye; 2 Department of Medical Pharmacology, Faculty of Medicine, Kahramanmaraş Sütçü İmam University, Kahramanmaraş, Türkiye

**Keywords:** congenital malformations, drug safety, glucagon-like peptide-1 receptor agonists, meta-analysis, perinatal outcomes, pregnancy, systematic review, tirzepatide

## Abstract

**Background:**

To evaluate maternal, fetal, and neonatal outcomes after preconception or gestational exposure to glucagon-like peptide-1 receptor agonists and dual glucagon-like peptide-1/glucose-dependent insulinotropic polypeptide receptor agonists, and to assess the reproducibility of reported safety signals.

**Methods:**

Six databases plus a drug-level safety search were searched through 16 July 2026. Exposure was classified into four clinically defined time windows. Independent studies or data sources were the analytic unit; one primary comparator was selected per study, and odds ratios and risk ratios were synthesized separately. Random-effects meta-analysis, timing-stratified and dominant-study sensitivity analyses, Newcastle-Ottawa Scale assessment, and GRADE certainty assessment were used.

**Results:**

Fourteen comparative data sources were included; 13 were quantitatively eligible and eight contributed to at least one pooled estimate. In the timing-specific analysis of major congenital malformations, four studies with verified periconceptional/first-trimester exposure yielded an odds ratio of 0.99 (95% confidence interval 0.77–1.27). A secondary pregnancy-period analysis that added one unadjusted, trimester-unspecified cohort yielded 1.00 (0.94–1.06); that cohort contributed 93.5% of the weight. Pooled preterm birth, small-for-gestational-age birth, and stillbirth analyses showed no statistically significant differences. Preeclampsia and other sparse outcomes were not pooled. Certainty was very low for all key outcomes.

**Conclusion:**

Current evidence does not demonstrate a clear or consistent increase in major congenital malformation risk, but very low certainty, imprecision, residual confounding, heterogeneous comparators, sparse agent-specific data, and uncertain exposure timing preclude conclusions of safety. Reported preeclampsia and renal or urinary signals have not been independently replicated.

## Introduction

Use of glucagon-like peptide-1 (GLP-1) receptor agonists has expanded rapidly among women of reproductive age, increasingly for indications other than diabetes. In an Australian cohort of 1,635,684 women, 90.5% of those initiating therapy in 2022 did not have type 2 diabetes and 2.2% became pregnant within 6 months of the first prescription (Thapaliya et al., 2025). Unintended exposure is therefore foreseeable; 164 unintended pregnancies were reported in clinical development programs including 32,598 women (Parker et al., 2025).

Interpretation is complicated by baseline differences between treated and comparator populations. Major congenital malformation prevalence is higher among infants of women with type 2 diabetes than in the general population (Cesta et al., 2024), and exposed women often have substantially higher body mass index than insulin-treated or unexposed comparators (Kolding et al., 2025; Hviid et al., 2026). Confounding by indication is therefore central.

Recent syntheses have reached discordant conclusions, ranging from lower maternal or preterm-birth risks to renal or urinary malformation signals driven largely by one unadjusted cohort (Kodali et al., 2026; Hakim et al., 2026; Uysal et al., 2026; Armstrong et al., 2025; Ozbek et al., 2026; Kuitunen, 2024). These discrepancies largely reflect exposure timing, comparator selection, and repeated weighting of overlapping or dominant data sources.

We therefore classified exposure into clinically defined time windows, separated effect measures, selected one primary comparator per independent study or data source, tracked source overlap, and examined dominant studies with leave-one-out analyses. The objective was to estimate pregnancy and perinatal associations while distinguishing independent replication from repeated pooling of the same evidence.

## Methods

### Protocol and transparency

The review methods were also informed by the JBI Manual for Evidence Synthesis (Aromataris et al., 2024). The review followed PRISMA 2020 and MOOSE (Page et al., 2021; Stroup et al., 2000). Searching, selection, initial extraction, an initial comparison-level synthesis, and an initial draft preceded registration. The protocol and amendment log were therefore deposited retrospectively on OSF on 30 July 2026 (Open-Ended Registration; embargo through 30 July 2027) and are not presented as prospective preregistration (https://osf.io/tr8e4). The original analysis used comparison-level pooling, allowed rare-outcome approximation across effect measures, and did not fully separate trimester-unspecified exposure. Before submission, corrective reanalysis adopted independent studies/data sources, one clinically prioritized comparator, effect-measure separation, Lewin as Window 3–4, and timing-, comparator-, dominance-, and rounding-sensitivity analyses. These changes addressed unit-of-analysis errors, incompatible estimands, and uncertain exposure timing; each change and rationale is dated in the OSF amendment log.

### Eligibility criteria

Comparative randomized, cohort, and case-control studies reporting maternal, fetal, or neonatal outcomes after preconception or gestational exposure to semaglutide, liraglutide, dulaglutide, exenatide, lixisenatide, albiglutide, or tirzepatide were eligible. Class-level synthesis was pragmatic: agents share GLP-1 receptor agonism, including tirzepatide’s GLP-1 component, but data were too sparse for agent-specific pooling. The grouping does not imply identical fetal effects; differences in structure, half-life, placental transfer, indication, and co-agonism were treated as indirectness. Case reports/series, non-human studies, unverifiable timing, and non-extractable abstracts were excluded. A propensity-score-matched cohort without a numerical estimate was retained narratively (Hanif et al., 2025). Exposure had to map to a predefined or combined window (Table 1; Supplementary Table S1). PICOS comprised pregnant or periconceptional women; GLP-1 or dual GLP-1/GIP agonist exposure; active, discontinuation, or unexposed comparators; maternal, fetal, or neonatal outcomes; and comparative randomized, cohort, or case-control designs.

**TABLE 1:** Exposure windows used in the corrective reanalysis and study assignment.

Window	Definition	Studies
1. Distant pre-pregnancy	Explicitly prepregnancy exposure ending >90 days before conception	Banerjee et al. (2026), planned-discontinuation cohort (use 6–18 months before the index encounter, discontinuation >90 days before)
2. Recent pre-pregnancy	Prepregnancy-only exposure ending 1–89 days before conception; no gestational exposure	Banerjee et al. (2026), abrupt-discontinuation cohort
1–2 combined	Definitions covering window 1 without excluding window 2	Imbroane et al. (2025), weight in window 1; Pondugula et al. (2026), pre-pregnancy stratum
3. Periconceptional/ first trimester	Exposure from day −90 through the end of the first trimester; day −90 assigned here	Cesta et al. (2024); Dao et al. (2024); Kolding et al. (2025); Hviid et al. (2026); Broe et al. (2025); Chou et al. (2026); Parker et al. (2025); Pondugula et al. (2026), within-pregnancy stratum
4. Continuation in pregnancy	Continued in the first trimester and beyond	Brown et al. (2026)
3–4 combined	Gestational exposure; trimester not separated	Lewin et al. (2026)
1–3 mixed	Estimates not separating pre-pregnancy from in-pregnancy exposure; excluded from primary analyses	Maya et al. (2025), weight in window 2; Pondugula et al. (2026), secondary outcomes; Hanif et al. (2025)

The non-interchangeability of Windows 1 and 2 was demonstrated within a single data source, where the GDM, estimate rose from RR, 0.97 to 1.54 according to discontinuation timing (Banerjee et al., 2026). GDM, gestational diabetes mellitus; RR, risk ratio.

### Search strategy, study selection, and data extraction

PubMed/MEDLINE, Embase, Web of Science, Scopus, the Cochrane Library, and Reprotox were searched with class-level and individual-drug terms combined with pregnancy concepts; an additional drug-level search targeted congenital-malformation and prescription-safety studies. The final update was 16 July 2026. Reference lists of recent syntheses yielded two additional eligible cohorts (Chou et al., 2026; Banerjee et al., 2026). E.M. performed the initial title/abstract screening. A.N.M. independently reviewed the study-selection decisions, including all full-text eligibility assessments and documented full-text exclusions. Differences were resolved through source re-review and consensus; no third adjudicator was required. Data were extracted by E.M. and independently verified by A.N.M.; all 14 comparative sources were confirmed without correction. Back-calculated event counts and rounding scenarios are reported in Supplementary Table S2. Supplementary Appendix S1 reconstructs the database-specific syntax from retained documentation; verbatim historical platform exports were not preserved.

### Exposure windows and comparator framework

Exposure was classified using a hierarchical, mutually exclusive study-level rule. Window 1 comprised explicitly prepregnancy exposure ending >90 days before conception. Window 2 comprised explicitly prepregnancy-only exposure ending 1–89 days before conception, with no reported gestational exposure. Day −90 was assigned to Window 3. Window 3 comprised exposure on or after day −90 through the end of the first trimester, including source-defined periconceptional windows that did not separate preconception-only from postconception exposure. Window 4 comprised continuation beyond the first trimester. When a source combined periods that could not be separated, it was assigned to a labeled combined window rather than forced into a single interval. For congenital malformations, the timing-specific primary analysis was restricted to Window 3; Window 3–4 was included only in a secondary pregnancy-period sensitivity analysis. The reproducible study-by-study assignments and source anchors are reported in Supplementary Table S1.

Comparators were categorized as diabetes-based active comparators, same-drug discontinuers, or unexposed/general-population controls. Each study contributed one prespecified primary comparator using that hierarchy; alternatives were exploratory (Supplementary Table S3). Because groups may differ in diabetes severity, obesity, indication, concomitant treatment, and healthcare use, pooled estimates represent heterogeneous study-specific contrasts, not one uniform causal effect.

## Outcomes

Outcomes included hypertensive disorders of pregnancy, preeclampsia, gestational diabetes, cesarean delivery, gestational weight gain, pregnancy loss, live birth, elective termination, stillbirth, major congenital malformations, cardiac and renal/urinary malformations, preterm birth, small- and large-for-gestational-age birth, and neonatal hypoglycemia, jaundice, and respiratory distress.

### Risk of bias and certainty of evidence

Observational studies were assessed independently by E.M. and A.N.M. with the Newcastle-Ottawa Scale (NOS) (Wells et al., 2026). NOS was selected because the evidence base consisted predominantly of cohort studies and it permitted a consistent study-level assessment of selection, comparability, and outcome ascertainment across the full set. RoB 2 is designed for randomized trials and was not applicable to this observational evidence base (Sterne et al., 2019); ROBINS-I was considered but was not used because the review had already implemented duplicate NOS assessment and the available aggregate reports did not support a reliable domain-complete ROBINS-I evaluation for every source (Sterne et al., 2016). NOS scores of 7–9, 5–6, and < = 4 were classified as low, moderate, and high risk, respectively; Comparability = 0 or Outcome < = 1 forced a high-risk judgment. Overall categories agreed for all 14 studies; three domain-level discrepancies were resolved by source re-review and consensus without a third adjudicator (Supplementary Tables S4, 5). Certainty was graded separately for each outcome using GRADE, with explicit reasons for downgrading reported in Supplementary Table S6 (Guyatt et al., 2008).

### Statistical analysis

Analyses were performed in R 4.4.2 with the meta and metafor packages. Random-effects models used Hartung-Knapp confidence intervals; when these were narrower than conventional random-effects intervals, the wider interval and corresponding p value were reported (DerSimonian and Laird, 1986; Hartung and Knapp, 2001). Primary pools were effect-measure specific: odds ratios were combined only with odds ratios and source-reported adjusted risk ratios were retained separately as study-level estimates. Raw-event odds ratios were calculated from 2 × 2 tables only when the source design and reporting supported an unpaired marginal comparison. Pooling required at least three independent studies on the same effect scale. A study contributing >75% of the weight triggered leave-one-out analysis; zero cells received a 0.5 continuity correction. For major congenital malformations, Window 3 formed the timing-specific primary analysis and the addition of Window 3–4 formed a secondary pregnancy-period sensitivity analysis. Restricted maximum likelihood and Paule-Mandel estimators were sensitivity analyses. Publication bias was not assessed quantitatively because no outcome had > = 10 independent non-overlapping comparisons.

For Cesta, the source-reported adjusted major-malformation risk ratio was retained at study level; its crude event-count odds ratio was also entered in the OR-specific synthesis because the published marginal counts supported that estimand (Cesta et al., 2024). Chou used 1:4 propensity-score matching with exact matching on maternal age group, infant year, and infant sex, followed by log-link Poisson generalized estimating equations. Its propensity score included demographics, lifestyle factors, maternal comorbidities, obstetric comorbidity, healthcare utilization, antihypertensive and lipid-modifying therapies, and concomitant antidiabetic medications (Chou et al., 2026). The source-reported adjusted risk ratio was therefore retained as the design-consistent study-level estimate. The simple MCM event-count odds ratio was not pooled because it ignores matched-set correlation and does not reproduce the source model; this decision was based on analytic compatibility, not on the direction of the estimate. Fewer than three adjusted risk ratios were available on a common scale, so adjusted estimates were not meta-analyzed. Potential source overlap was assessed by database, calendar period, exposed arm, comparator, and outcome, and shared participants were not entered together in a primary pool.

### Use of generative AI

OpenAI ChatGPT (GPT-5.6 Sol; OpenAI, San Francisco, CA, United States) was used for English-language translation, editorial refinement, document formatting, and technical assistance in preparing reproducibility materials. All source data, analytical code, statistical outputs, references, and scientific interpretations were reviewed and verified by the authors, who take full responsibility for the work.

## Results

### Study selection and characteristics

The searches identified 2,312 records; 1,044 duplicates were removed and 1,268 records were screened. Forty-nine database-route full texts were assessed and 37 excluded: no comparator (n = 9), no pregnancy outcome (n = 5), unverifiable exposure window (n = 4), no extractable data (n = 4), case report or series (n = 6), duplicate publication (n = 3), insufficient conference abstract (n = 3), or non-human study (n = 3). Twelve comparative sources were identified through database searching and two through citation searching, yielding 14 included sources; 13 were quantitatively eligible and eight contributed to at least one pool (Figure 1). Reported exposed-group sizes summed to at least 46,341 pregnancies but should not be interpreted as unique women because data sources may overlap. Study characteristics and each source’s contribution to narrative, study-level, or pooled synthesis are provided in Table 2.

**FIGURE 1:**
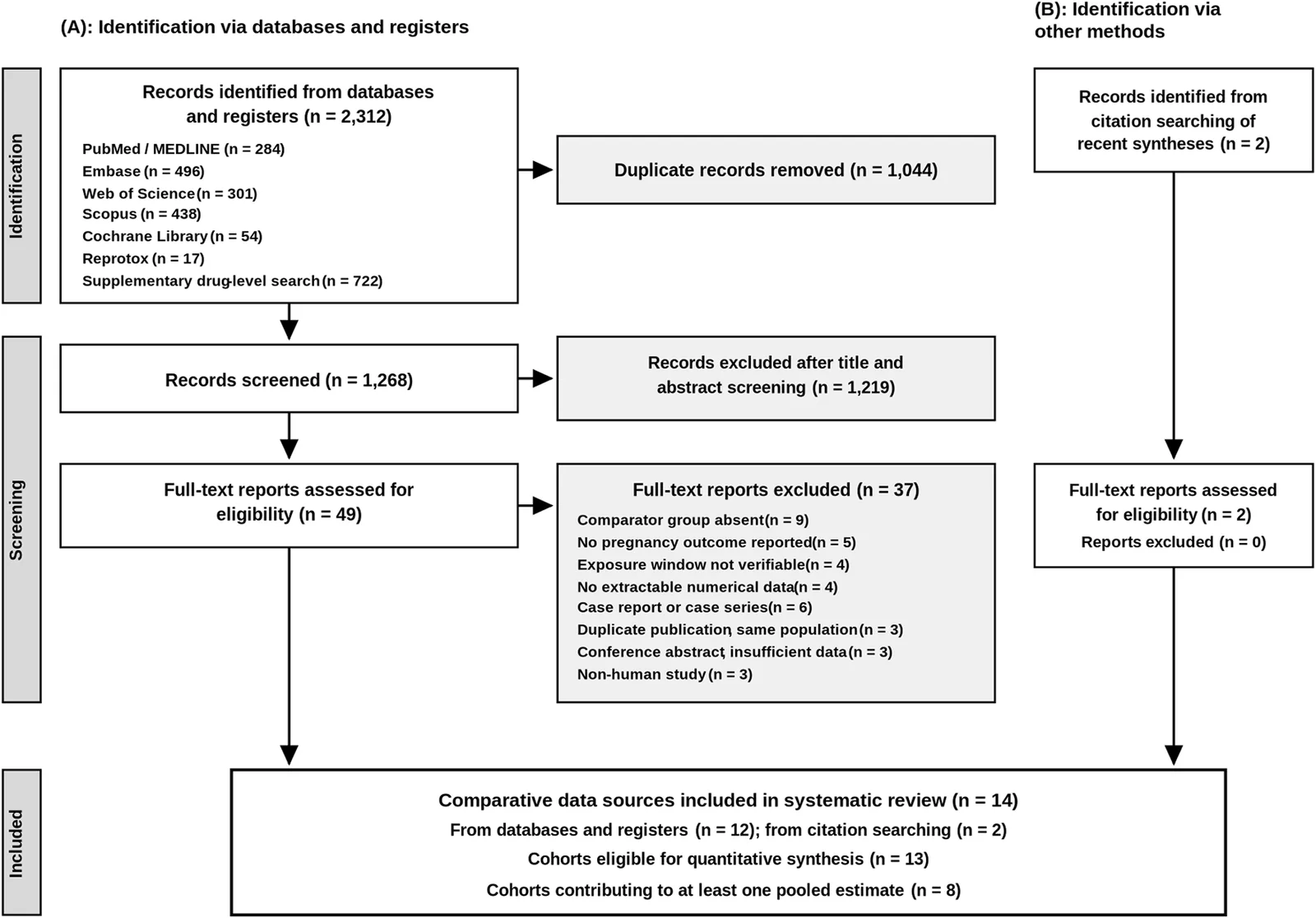
PRISMA 2020 flow diagram of study identification, screening, eligibility assessment, and inclusion. Of the 14 comparative data sources included, 13 were eligible for quantitative synthesis and 8 contributed to at least one pooled estimate under the study-level pooling threshold of three independent studies. GLP-1 RA, glucagon-like peptide-1 receptor agonist; PRISMA, Preferred Reporting Items for Systematic Reviews and Meta-Analyses.

**TABLE 2:** Characteristics and synthesis contribution of the 14 included comparative data sources.

Study	Data source	Window	Exposed	Primary comparator used	Use in synthesis
Cesta et al. (2024)	Nordic registers 2009–2020 + US MarketScan 2012–2021 + israeli Maccabi 2009–2020	3	938	Insulin (no ADM other than metformin) 5,078	MCM timing-specific OR analysis (crude OR); adjusted RR shown separately
Dao et al. (2024)	Six teratology information services	3	168	Diabetes reference, n = 156	MCM timing-specific, stillbirth, preterm birth and SGA OR analyses; other outcomes at study level
Broe et al. (2025)	Danish national screening	3	55	Same-drug prepregnancy discontinuers	MCM timing-specific OR analysis
Hanif et al. (2025)	Federated electronic health record network (TriNetX)	1–3 mixed	NR for outcome synthesis	Propensity-score-matched comparator; numerical outcome data not reported	Narrative synthesis only; no point estimate or raw event count; excluded from quantitative synthesis
Imbroane et al. (2025)	US TriNetX	1–2	4,267	4,267	HDP at study level; not pooled
Kolding et al. (2025)	Danish regional	3	32	Insulin, n = 547	MCM timing-specific OR analysis; other outcomes at study level
Maya et al. (2025)	US single academic health system (2016–2025)	1–3 mixed	448	PS 1:3, 1,344	Study level
Parker et al. (2025)	FDA/EMA files	3	≥ 111	Placebo ≥38	Stillbirth primary OR pool; pregnancy outcomes at study level
Banerjee et al. (2026)	Multinational TriNetX (2015–2025)	1 and 2	735	PS 1:1 (including BMI and HbA1c)	Study level; basis of the window rule
Brown et al. (2026)	US MarketScan (2011–2024)	4	3,572	Discontinuers (within cohort)	Study level
Chou et al. (2026)	Taiwan national (BCA 2014–2022; NHI 2013–2022)	3	160	Insulin (no GLP-1 RA; other ADMs allowed), PS 1:4, 606	MCM adjusted RR retained at study level; simple aggregate OR not pooled because it does not preserve the matched GEE analysis; SGA and stillbirth OR analyses
Hviid et al. (2026)	Danish national	3	529 (295 liraglutide; 234 semaglutide)	Agent-specific propensity-score-matched controls	Preterm birth and SGA primary OR pools; LGA and preeclampsia at study level
Lewin et al. (2026)	US Epic Cosmos (May 2022 – April 2025)	3–4 combined	35,083	PGDM without GLP-1 RA 108,510	MCM secondary pregnancy-period sensitivity and preterm-birth OR analysis; preeclampsia at study level; > 75% sensitivity
Pondugula et al. (2026)	US single center	Variable	243	PGDM 175; obese 200	HDP and GWG at study level; not pooled

PS, propensity score; ADM, antidiabetic medication; PGDM, pregestational diabetes mellitus; GWG, gestational weight gain; MCM, major congenital malformation; SGA, small for gestational age; LGA, large for gestational age; HDP, hypertensive disorders of pregnancy; BCA, birth certificate application registry; NHI, national health insurance; NR, not reported/not extractable for outcome synthesis. Concomitant insulin was permitted in the exposed arms of the multinational registry cohort and the Taiwanese cohort; in the former, 38.5%–62.2% of exposed pregnancies had insulin co-prescription (see Methods: Exposure definitions and comparator hierarchy). Hanif et al. (2025) was retained in the systematic review but excluded from quantitative synthesis because no point estimate or raw event count was reported for any outcome. Thus, 14 comparative data sources were included, 13 were quantitatively eligible, and 8 contributed to at least one pooled estimate.

Agent-specific evidence was limited. Hviid reported 295 liraglutide-exposed and 234 semaglutide-exposed pregnancies with separate agent-level estimates for selected outcomes, and Kolding reported 32 semaglutide-exposed pregnancies. Dao reported the exposed cohort by agent, but outcome-event counts were not sufficiently reported by individual agent for comparative pooling. Most remaining sources reported class-level exposure or did not provide agent-specific outcome counts; extractable comparative tirzepatide-specific pregnancy outcomes were unavailable. Consequently, no agent-specific meta-analysis was performed, and class-level estimates should not be interpreted as establishing safety for semaglutide, liraglutide, tirzepatide, or any other individual agent (Supplementary Table S8).

### Risk of bias

Final consensus classified 10 studies as low, one as moderate, and three as high risk (Supplementary Tables S4, 5). Kolding and Lewin were high risk primarily because of inadequate comparability or confounder control, and Hanif because no numerical outcome estimate was available. In the secondary pregnancy-period MCM analysis, high-risk Kolding and Lewin contributed 93.7% of the weight; Lewin alone contributed 93.5%.

### Major congenital malformations

The timing-specific major-malformation analysis included four independent studies with verified periconceptional/first-trimester exposure (Cesta et al., 2024; Kolding et al., 2025; Broe et al., 2025; Dao et al., 2024) and yielded an odds ratio of 0.99 (95% CI 0.77–1.27). A secondary pregnancy-period sensitivity analysis added Lewin, whose gestational exposure timing was not trimester-resolved, and yielded 1.00 (0.94–1.06; p = 0.99; I^2^ = 0%) (Table 3; Figure 2). Lewin lacked covariate adjustment and contributed 93.5% of the secondary analysis weight. Accordingly, the apparently precise 1.00 estimate is not interpreted as a general or first-trimester risk estimate; the timing-specific analysis is materially less precise.

**TABLE 3:** Pooled and unpooled outcomes by exposure window.

Outcome	Independent studies	Window	OR (95% CI); p	Heterogeneity	GRADE
Panel A. Pooled outcomes (≥3 independent studies)					
Major congenital malformation (timing-specific primary)	4	3	0.99 (0.77–1.27)	I^2^ = 0%	Very low
Major congenital malformation (pregnancy-period sensitivity)	5	3 + 3–4	1.00 (0.94–1.06); 0.99	I^2^ = 0%	Very low
Preterm birth	3	3 + 3–4	1.16 (0.47–2.82); 0.56	I^2^ = 73.6%; τ^2^ = 0.038	Very low
SGA	3	3	0.99 (0.77–1.26); 0.91	I^2^ = 0%	Very low
Stillbirth	3	3	0.93 (0.04–22.9); 0.93	I^2^ = 47.1%; τ^2^ = 0.918	Very low

Outcome	Independent studies	Window	Study-level estimates	Note
Panel B. Outcomes not pooled (fewer than three independent studies)				
Preeclampsia	2	3 + 3–4	Lewin 1.12 (1.08–1.16), unadjusted; Hviid within-study combined 1.08 (0.83–1.42)	Not pooled; see results: Other pregnancy and perinatal outcomes
Hypertensive disorders of pregnancy	2	1–2	Imbroane and Pondugula, pre-pregnancy stratum	Not pooled; see results: Other pregnancy and perinatal outcomes
LGA	2	3	Hviid; Dao: 3.38 vs. obese reference, 0.70 vs. diabetes reference	Not pooled; see results: Other pregnancy and perinatal outcomes
Spontaneous pregnancy loss	2	3	Dao; Parker	Not pooled; see results: Other pregnancy and perinatal outcomes
Live birth	2	3	Dao; Kolding	Not pooled; see results: Other pregnancy and perinatal outcomes
Elective termination	2	3	Dao: aHR 3.89 (1.48–10.2) vs. diabetes reference, 1.39 (0.66–2.93) vs. obese reference; Parker	Not pooled; see results: Other pregnancy and perinatal outcomes

Random-effects estimates with Hartung-Knapp confidence intervals; where the Hartung-Knapp interval was narrower than the conventional random-effects interval, the wider interval and corresponding p value were reported. ORs, were pooled only with ORs, and reported RRs, were not relabeled as ORs. Raw-count ORs, were used only when compatible with the source design; adjusted native-scale estimates were retained separately. Each independent study/data source contributed one comparator selected by the corrective-reanalysis hierarchy (diabetic active comparator > same-drug discontinuers > unexposed/general population). The MCM, Window 3 row is the timing-specific primary analysis; the 3 + 3–4 row adds the trimester-unspecified Lewin cohort as a secondary pregnancy-period sensitivity. Lewin contributed 93.5% of that sensitivity analysis weight. The dominant-cohort rule (weight >75%) also applied to SGA (Hviid et al., 88.1%; after exclusion OR, 0.93, 0.39–2.24). Study-level risk of bias is shown in Supplementary Tables S4, 5 and GRADE, in Supplementary Table S6. OR, odds ratio; CI, confidence interval; RR, risk ratio; aHR, adjusted hazard ratio.

**FIGURE 2:**
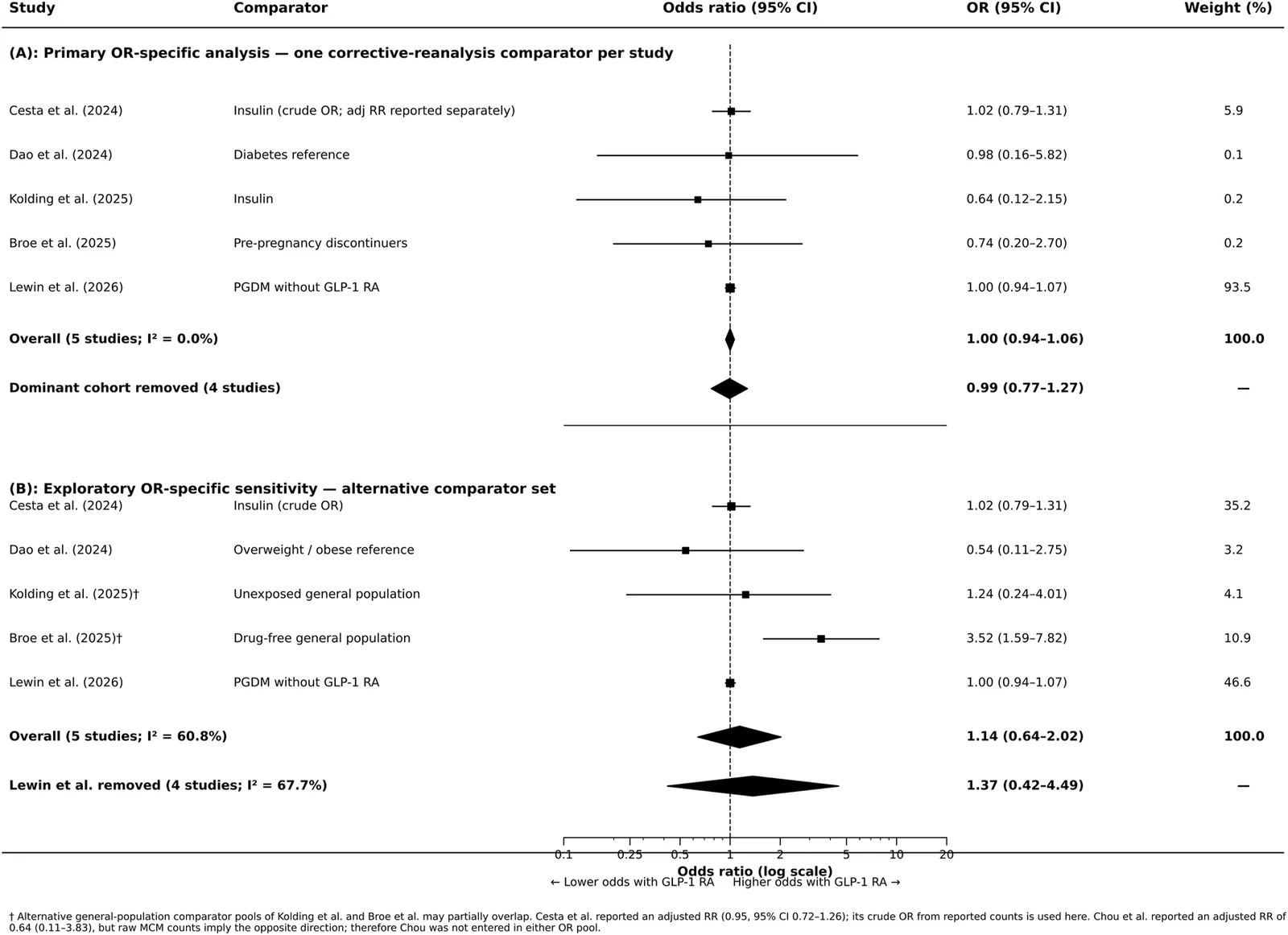
Major congenital malformation after exposure to GLP-1 receptor agonists. Panel A shows the OR-specific analysis with one comparator per independent study selected by the corrective-reanalysis hierarchy. The timing-specific Window 3 estimate excludes Lewin et al.; adding its trimester-unspecified Window 3–4 cohort yields the secondary pregnancy-period estimate. Panel B shows an exploratory alternative-comparator sensitivity. Cesta et al., 2024 contributed a crude OR from reported marginal counts, with its adjusted RR retained separately. For Chou et al., 2026 the source-reported propensity-score-matched GEE RR was retained as the design-consistent estimate; a simple OR from aggregate matched-sample counts was not pooled because it ignores matched-set correlation. CI, confidence interval; OR, odds ratio; RR, risk ratio; GEE, generalized estimating equation; PGDM, pregestational diabetes mellitus.

Comparator choice materially affected estimates. Cesta reported adjusted RR 0.95 versus insulin and crude OR 1.02 (Cesta et al., 2024). Dao reported adjusted ORs 0.98 versus a diabetes reference and 0.54 versus an overweight/obese reference (Dao et al., 2024); Kolding yielded 0.64 versus insulin and 1.24 versus an unexposed population (Kolding et al., 2025); Broe yielded 3.52 versus a drug-free population and 0.74 versus prepregnancy discontinuers, the primary comparator (Broe et al., 2025). Chou reported a propensity-score-matched GEE RR of 0.64 (0.11–3.83); simple counts (1/160 vs. 1/606) yield marginal OR 3.81, but that unpaired calculation does not preserve the matched analysis and was not substituted for the adjusted estimate (Chou et al., 2026). Alternative comparators produced pregnancy-period OR 1.14 (0.64–2.02; I^2^ = 60.8%); without Lewin, 1.37 (0.42–4.49) (Supplementary Table S3). These clinically different contrasts were not interpreted as one causal comparison.

Cardiac malformations were numerically reported in only one study (Cesta et al., 2024). The only numerical renal-category estimate was an unadjusted urinary-malformation odds ratio of 1.25 (1.05–1.49) from Lewin; rounding scenarios ranged from 1.00 to 1.57, with three of nine intervals including 1 (Supplementary Table S2) (Lewin et al., 2026). Similar renal or urinary estimates in previous meta-analyses were driven by the same dominant cohort and therefore do not constitute independent replication (Hakim et al., 2026; Uysal et al., 2026).

### Other pregnancy and perinatal outcomes

Stillbirth was pooled across three independent studies and yielded an odds ratio of 0.93 (95% CI 0.04–22.9; I^2^ = 47.1%) (Parker et al., 2025; Chou et al., 2026; Dao et al., 2024). Preterm birth yielded 1.16 (0.47–2.82; I^2^ = 73.6%) (Hviid et al., 2026; Lewin et al., 2026; Dao et al., 2024), and small-for-gestational-age birth yielded 0.99 (0.77–1.26; I^2^ = 0%); after removal of the dominant Hviid study, the latter was 0.93 (0.39–2.24) (Hviid et al., 2026; Chou et al., 2026; Dao et al., 2024). Spontaneous loss, live birth, elective termination, and large-for-gestational-age birth did not meet the pooling threshold (Table 3).

Preeclampsia was reported by two independent studies and was not pooled (Figure 3). Lewin yielded an unadjusted odds ratio of 1.12 (1.08–1.16), whereas the within-study combined Hviid estimate was 1.08 (0.83–1.42) (Hviid et al., 2026; Lewin et al., 2026). The signal was considered exploratory because it was not independently reproduced and residual confounding remained. Hypertensive disorders of pregnancy, gestational diabetes, and cesarean delivery also did not meet the pooling threshold. Neonatal morbidity was reported in one study, and gestational-weight-gain measures were too heterogeneous for pooling (Kolding et al., 2025; Banerjee et al., 2026; Pondugula et al., 2026; Imbroane et al., 2025; Maya et al., 2025).

**FIGURE 3:**
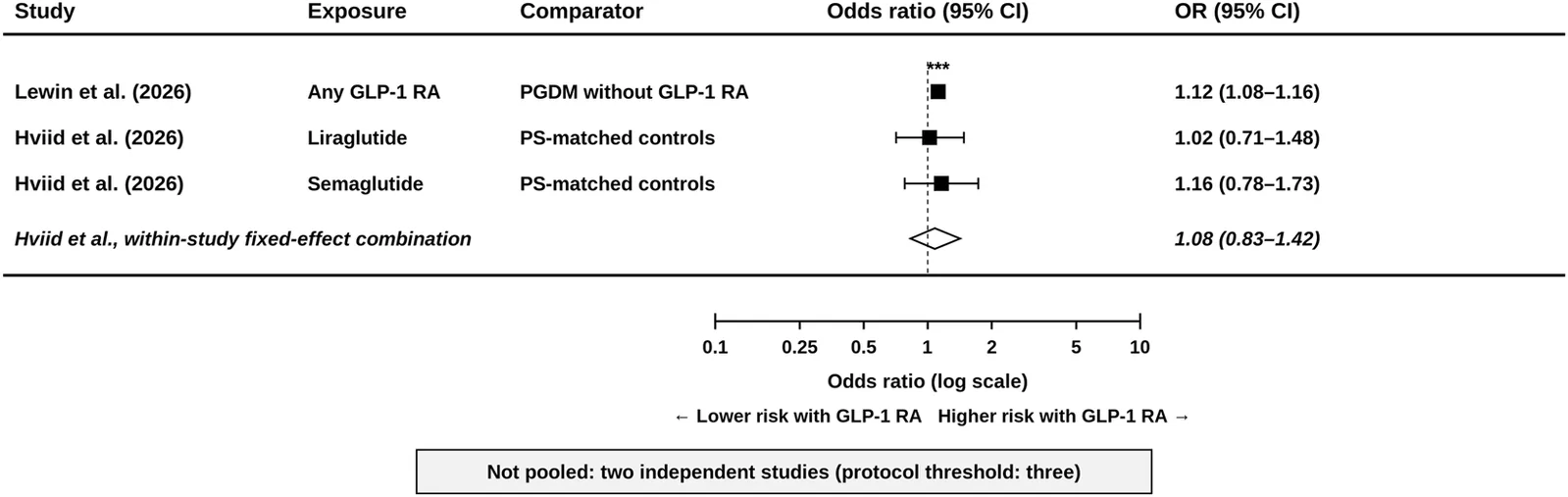
Study-level estimates for preeclampsia after pregnancy-period exposure to GLP-1 receptor agonists. Estimates were not pooled because only two independent studies were available. The agent-specific comparisons from Hviid et al. (2026) are shown together with their within-study fixed-effect combination (open diamond). ***p < 0.001, unadjusted single-cohort estimate. CI, confidence interval; GLP-1 RA, glucagon-like peptide-1 receptor agonist; OR, odds ratio; PGDM, pregestational diabetes mellitus; PS, propensity score.

### Certainty and relation to previous syntheses

None of the pooled outcomes was statistically significant. Certainty was very low because of observational design, residual confounding, comparator/timing indirectness, sparse events, dominance by high-risk unadjusted data, or imprecision (Supplementary Table S6). Thus, non-significance is not evidence of safety. Earlier renal/urinary signals came largely from the same unadjusted cohort, whereas syntheses reporting lower maternal or preterm-birth risks weighted prepregnancy exposure substantially (Table 4) (Kodali et al., 2026; Hakim et al., 2026; Uysal et al., 2026; Armstrong et al., 2025; Ozbek et al., 2026).

**TABLE 4:** Comparison with previous systematic reviews and meta-analyses.

Synthesis	Studies	MCM	Cardiac	Renal/urinary	Preterm birth	Stillbirth
Hakim et al. (2026)	22	1.19 (0.72–1.98)	0.88 (0.53–1.44)	1.23 (1.09–1.39)	1.11 (0.84–1.47)	—
Kodali et al. (2026)	4	1.08 (0.86–1.37)	—	—	0.66 (0.53–0.82)	—
Armstrong et al. (2025)	9*	p = 0.79	p = 0.03 (reduction)	—	p = 0.62	p = 0.09
Uysal et al. (2026)	7	1.39 (0.73–2.65)^†^	0.92 (0.67–1.27)	1.24 (1.05–1.47)	1.17 (0.79–1.72)	2.17 (0.95–4.91)^‡^
Ozbek et al. (2026)	36	No increase	—	—	Heterogeneous	No increase
Kuitunen (2024)	2	No signal of increase (not pooled)	Not pooled	—	—	—
This study (window 3 primary)	14 included; 13 quantitative	0.99 (0.77–1.27); pregnancy-period sensitivity 1.00 (0.94–1.06)§	Not pooled	Single cohort 1.25 (1.05–1.49)	1.16 (0.47–2.82)§	0.93 (0.04–22.9)§

Values are odds ratios (ORs) with 95% confidence intervals unless otherwise stated. Comparability differs across syntheses: Hakim pooled broadly defined periconceptional/gestational exposure using ORs; Kodali combined preconception and gestational exposure using ORs; Armstrong included preconception or pregnancy exposure and reported pooled ORs, or p values; Uysal reported a first-trimester MCM OR, and pregnancy-period ORs, for other outcomes; Ozbek combined heterogeneous windows and reported qualitative or heterogeneous results where effect sizes were unavailable; Kuitunen evaluated first-trimester exposure without pooled MCM, or cardiac estimates. Our renal/urinary estimate is an unadjusted single-cohort OR (Lewin et al., 2026). *Armstrong reported 6 studies in the abstract and 9 in study selection; unavailable preview effect sizes are shown as p values.

^†^ First-trimester MCM, estimate (k = 5; I2 = 52%); any congenital malformation was a separate outcome.

^‡^ Regulatory-data arms were reversed in that synthesis and are not directly comparable. §Independent-study analysis with one primary comparator per study; Window 3 is primary for MCM, and the 3 + 3–4 estimate is a secondary pregnancy-period sensitivity.

## Discussion

### Principal findings

This review of 14 comparative data sources found no clear or consistent increase in major congenital malformations after periconceptional/first-trimester GLP-1-based therapy exposure, but the timing-specific estimate was imprecise. Adding one large unadjusted cohort with unresolved trimester timing produced a near-null, apparently precise pregnancy-period estimate dominated by that cohort (93.5% weight). Pooled preterm-birth, small-for-gestational-age, and stillbirth analyses were also not statistically significant. Given very low certainty and residual confounding, these findings neither establish safety nor exclude clinically important harm.

The nominal preeclampsia association was not reproduced in an independent matched cohort, and the renal/urinary signal traced to one dominant unadjusted source and was sensitive to rounding assumptions. Repetition of the same estimate across meta-analyses should therefore not be mistaken for biological replication.

### Exposure timing, comparators, and clinical interpretation

Exposure timing is clinically consequential. Trimester-unspecified gestational exposure cannot be interpreted as a first-trimester estimate, whereas prepregnancy exposure may reflect residual pharmacologic effects and metabolic rebound after withdrawal. Comparator choice likewise changes the causal question: active diabetes comparators partly balance underlying disease burden; same-drug discontinuers address continuation versus cessation but may be affected by reasons for stopping; and general-population controls may differ markedly in diabetes severity, obesity, treatment indication, concomitant therapy, and healthcare use. The marked comparator dependence of malformation estimates illustrates why clinically incompatible control groups should not be collapsed into a single causal interpretation.

## Clinical implications

Current evidence does not support treating inadvertent GLP-1-based therapy exposure, by itself, as proof of teratogenicity or as an indication for pregnancy termination. Conversely, lack of statistical significance should not be equated with established safety. Counseling should incorporate exposure timing, agent, diabetes and obesity severity, concomitant therapy, and patient preferences. Associations after proximal or abrupt discontinuation also support careful preconception planning and continued weight and glycemic surveillance, although causal management recommendations require prospective data.

## Strengths and limitations

Strengths include explicit time-window classification, separate coding of trimester-unspecified exposure, independent review of study-selection and full-text eligibility decisions, use of the independent study or data source as the analytic unit, one primary comparator per study, effect-measure-specific pooling, explicit overlap assessment, timing-, dominant-study-, comparator-, and rounding-sensitivity analyses, outcome-level GRADE, independent data verification, and duplicate NOS assessment. Limitations are the exclusively observational evidence, retrospective rather than prospective registration, *post hoc* corrective analytic changes, heterogeneous exposure, outcome, and comparator definitions, residual confounding, lack of three independent adjusted estimates on a common scale for any outcome, dominant unadjusted cohorts, possible database overlap, sparse agent-specific data, and inability to assess publication bias. Pooling GLP-1 receptor agonists with tirzepatide was pragmatic and cannot establish a uniform class effect because molecular structure, half-life, placental transfer, indication, and GIP co-agonism may differ. These limitations underpin the very low certainty ratings.

Future studies should anchor exposure to conception, report trimester-specific continuation, define indication and diabetes subtype, measure body mass index, glycated hemoglobin, weight change and concomitant insulin, and cross-reference overlapping databases. Active-comparator, target-trial-emulation, and prospective teratology designs should be prioritized.

Current very-low-certainty comparative evidence does not demonstrate a clear or consistent increase in major congenital malformation risk after periconceptional/first-trimester exposure to GLP-1-based therapies, but it cannot establish safety or exclude clinically important harm. Preeclampsia and renal/urinary signals have not been independently replicated. Inadvertent exposure alone is not evidence supporting pregnancy termination; decisions should be individualized while better prospective, timing-specific, comparator-aligned, and agent-specific data are generated.

## Data Availability

The original contributions presented in the study are included in the article/Supplementary Material, further inquiries can be directed to the corresponding author.
