# Supplementary material for: Pregnancy and perinatal outcomes following exposure to GLP-1 and dual GLP-1/GIP receptor agonists: a time-window-stratified systematic review and meta-analysis
Source: Front Pharmacol. 2026 Oct 16;17:1994366. doi: 10.3389/fphar.2026.1994366 (PMC13719578; doi:10.3389/fphar.2026.1994366)
Supplement: Supplementary file 1 [file Table1.docx]

**PRISMA 2020 Checklist - Manuscript Location Map**

Pregnancy and Perinatal Outcomes Following Exposure to GLP-1 and Dual GLP-1/GIP Receptor Agonists: A Time-Window-Stratified Systematic Review and Meta-analysis

| **Item** | **Topic** | **Reported content** | **Location** |
| --- | --- | --- | --- |
| 1 | Title | Identifies report as a systematic review and meta-analysis. | Title page/title. |
| 2 | Abstract | Structured summary. | Abstract. |
| 3-4 | Rationale/objectives | Rationale and explicit objective. | Introduction. |
| 5 | Eligibility criteria | Design, exposure, comparator, outcomes and exclusions. | Methods - Eligibility criteria. |
| 6 | Information sources | Databases, other sources, final date. | Methods - Search strategy; Appendix S1. |
| 7 | Search strategy | Database-specific strategy. | Appendix S1; transparently reconstructed from retained documentation because verbatim historical query exports were not preserved. |
| 8 | Selection process | Eligibility process. | Methods - Search strategy, study selection, and data extraction. |
| 9 | Data collection | Extraction and independent verification. | Methods - Search strategy, study selection, and data extraction. |
| 10 | Data items | Outcomes and study/exposure/comparator variables. | Methods - Outcomes; Table 1; Table 2; Supplementary Table S1. |
| 11 | Risk of bias | Duplicate NOS assessment and consensus. | Methods - Risk of bias; Supplementary Tables S4-S5. |
| 12 | Effect measures | ORs/RRs. | Methods - Statistical analysis. |
| 13 | Synthesis methods | Preparation, models, heterogeneity, sensitivity analyses. | Methods - Statistical analysis; Supplementary Tables S2-S3. |
| 14 | Reporting bias | Planned threshold and non-assessment when k<10. | Methods - Statistical analysis. |
| 15 | Certainty | Outcome-level GRADE. | Methods - Risk of bias; Supplementary Table S6. |
| 16 | Study selection | Flow and exclusion reasons. | Results; Figure 1. |
| 17 | Characteristics | Included-study characteristics. | Table 2; Supplementary Table S1. |
| 18 | Risk of bias results | Study-level NOS. | Results; Supplementary Tables S4-S5. |
| 19 | Individual results | Study-level estimates/events. | Results; Supplementary Table S3. |
| 20 | Synthesis results | Pooled estimates, heterogeneity and sensitivity. | Results; Table 3; Figure 2; Supplementary Tables S2-S3. |
| 21 | Reporting biases | Not assessed quantitatively because k<10. | Methods/Results. |
| 22 | Certainty | GRADE ratings. | Results; Supplementary Table S6. |
| 23 | Discussion | Interpretation, limitations, implications. | Discussion. |
| 24 | Registration/protocol/amendments | Retrospective OSF registration disclosed. | Methods - Protocol and transparency. |
| 25 | Support | Funding. | Funding. |
| 26 | Competing interests | Conflict of interest. | Conflict of Interest. |
| 27 | Data/code availability | Availability statement. | Data Availability Statement. |
