# Supplementary material for: Pregnancy and perinatal outcomes following exposure to GLP-1 and dual GLP-1/GIP receptor agonists: a time-window-stratified systematic review and meta-analysis
Source: Front Pharmacol. 2026 Oct 16;17:1994366. doi: 10.3389/fphar.2026.1994366 (PMC13719578; doi:10.3389/fphar.2026.1994366)
Supplement: Supplementary file 2 [file Table2.docx]

**SUPPLEMENTARY MATERIAL**

This file contains Appendix S1 and Supplementary Tables S1-S8 cited in the main manuscript.

**Appendix S1. Reconstructed database-specific search strategy for reproducibility**

The retained project files document the databases, search concepts, agent terms, final search date, and database-specific record counts, but do not preserve the verbatim historical platform query histories. The syntax below reconstructs those documented concepts in database-appropriate form for reproducibility and should not be interpreted as a verbatim export of the original saved histories. The original PRISMA record counts remain those generated by the searches completed through July 16, 2026.

| **Database/source** | **Final search date** | **Reconstructed syntax / documented search framework** | **Records in original flow** |
| --- | --- | --- | --- |
| PubMed/MEDLINE | July 16, 2026 | (("Glucagon-Like Peptide-1 Receptor Agonists"[Mesh] OR "glucagon-like peptide-1 receptor agonist*"[tiab] OR GLP-1[tiab] OR GLP1[tiab] OR semaglutide[tiab] OR liraglutide[tiab] OR dulaglutide[tiab] OR exenatide[tiab] OR lixisenatide[tiab] OR albiglutide[tiab] OR tirzepatide[tiab]) AND ("Pregnancy"[Mesh] OR pregnan*[tiab] OR gestation*[tiab] OR maternal[tiab] OR fetal[tiab] OR foetal[tiab] OR congenital[tiab] OR malformation*[tiab] OR birth[tiab] OR neonatal[tiab])) | 284 |
| Embase | July 16, 2026 | ('glucagon like peptide 1 receptor agonist'/exp OR 'glucagon like peptide 1 receptor agonist':ti,ab,kw OR semaglutide:ti,ab,kw OR liraglutide:ti,ab,kw OR dulaglutide:ti,ab,kw OR exenatide:ti,ab,kw OR lixisenatide:ti,ab,kw OR albiglutide:ti,ab,kw OR tirzepatide:ti,ab,kw) AND ('pregnancy'/exp OR pregnan*:ti,ab,kw OR gestation*:ti,ab,kw OR maternal:ti,ab,kw OR fetal:ti,ab,kw OR congenital:ti,ab,kw OR malformation*:ti,ab,kw OR neonatal:ti,ab,kw) | 496 |
| Web of Science Core Collection | July 16, 2026 | TS=(("glucagon-like peptide-1 receptor agonist*" OR "GLP-1 receptor agonist*" OR GLP1 OR semaglutide OR liraglutide OR dulaglutide OR exenatide OR lixisenatide OR albiglutide OR tirzepatide) AND (pregnan* OR gestation* OR maternal OR fetal OR foetal OR congenital OR malformation* OR birth OR neonatal)) | 301 |
| Scopus | July 16, 2026 | TITLE-ABS-KEY(("glucagon-like peptide-1 receptor agonist*" OR "GLP-1 receptor agonist*" OR GLP1 OR semaglutide OR liraglutide OR dulaglutide OR exenatide OR lixisenatide OR albiglutide OR tirzepatide) AND (pregnan* OR gestation* OR maternal OR fetal OR foetal OR congenital OR malformation* OR birth OR neonatal)) | 438 |
| Cochrane Library | July 16, 2026 | ([mh "Glucagon-Like Peptide-1 Receptor Agonists"] OR "GLP-1 receptor agonist*":ti,ab,kw OR semaglutide:ti,ab,kw OR liraglutide:ti,ab,kw OR dulaglutide:ti,ab,kw OR exenatide:ti,ab,kw OR lixisenatide:ti,ab,kw OR albiglutide:ti,ab,kw OR tirzepatide:ti,ab,kw) AND ([mh Pregnancy] OR pregnan*:ti,ab,kw OR gestation*:ti,ab,kw OR congenital:ti,ab,kw OR malformation*:ti,ab,kw OR neonatal:ti,ab,kw) | 54 |
| Reprotox | July 16, 2026 | Agent-by-agent searches for semaglutide, liraglutide, dulaglutide, exenatide, lixisenatide, albiglutide, tirzepatide, and class-level GLP-1 receptor agonist terminology, with pregnancy/reproductive safety records reviewed for comparative human pregnancy outcomes. | 17 |
| Supplementary drug-level safety search | July 16, 2026 | Individual agent terms (semaglutide OR liraglutide OR dulaglutide OR exenatide OR lixisenatide OR albiglutide OR tirzepatide) combined with congenital-malformation and prescription-drug safety concepts (congenital OR malformation* OR birth defect* OR pregnancy outcome* OR prescription safety OR drug safety). | 722 |

Manual searching: study tables and reference lists of recent systematic reviews/meta-analyses were screened; two additional eligible cohorts (Chou et al., 2026 and Banerjee et al., 2026) were identified by this route.

**Supplementary Table S1. Study-level exposure definitions, pregnancy time anchors, and exposure-window assignments**

| **Study** | **Exposure definition (as reported in the source)** | **Pregnancy time anchor** | **Exposure ascertainment** | **Window** | **Notes** |
| --- | --- | --- | --- | --- | --- |
| Cesta et al. (2024) | At least one dispensing of a drug in the class from 90 days before the first day of the LMP to the end of the first trimester | First day of LMP | Prescription dispensing (national registers and claims data) | 3 | Concomitant antidiabetics, including insulin, permitted in the exposed arm (insulin co-prescription 38.5–62.2%). Adjustment limited to birth year, maternal age, obesity and country. Analysis restricted to live births. Fetal chromosomal anomalies and known teratogen exposure excluded at pregnancy level. MarketScan overlap with Brown et al. |
| Dao et al. (2024) | GLP-1 RA exposure in early pregnancy; two reference groups (diabetic; overweight/obese) | Not reported; gestational week at first contact used | Teratology information service interview, prospective | 3 | Of the two reference groups, the diabetic reference was the primary comparator; the overweight/obese reference was used in the sensitivity set (see Methods: Statistical analysis). Major defect assessment restricted to live births. |
| Kolding et al. (2025) | Semaglutide and/or insulin in the first trimester; up to 70 days after the estimated conception date (gestational age 12+0/7) | Estimated conception date | Electronic patient records, regional | 3 | Malformations classified according to EUROCAT. The number of exposed pregnancies (n = 32) precluded confounder adjustment. Danish source overlap with Hviid et al. and Broe et al. |
| Chou et al. (2026) | Any GLP-1 RA dispensing from 90 days before the first day of the LMP to the end of the first trimester (LMP + 90 days) | First day of LMP; derived by subtracting gestational age from the delivery date | Outpatient dispensing records, national health insurance | 3 | Concomitant antidiabetics, including insulin, permitted in the exposed arm; the comparator was an insulin group without GLP-1 RA but with other agents allowed. Pregnancy-level exclusions: multiple gestation (n = 221), chromosomal anomalies (n = 10), first-trimester teratogen exposure (n = 123). Propensity score matching 1:4. |
| Broe et al. (2025) | Prescription dispensing between the first day of the LMP and the end of the first trimester | First day of LMP; derived by subtracting gestational age from the delivery date | National prescription registry | 3 | Drug-level safety screening covering 115 drugs and more than 700 drug–outcome associations without multiplicity correction. The adjustment model does not include diabetes diagnosis. Danish source overlap. |
| Hviid et al. (2026) | Prescription dispensing within 8 weeks before or after the LMP; liraglutide and semaglutide analyzed separately | LMP | National Prescription Register | 3 | The window is narrower than Window 3 but lies entirely within it. Six propensity-score-matched comparison groups. Stillbirth counts not reported for confidentiality reasons. Danish source overlap with Kolding et al. and Broe et al. |
| Imbroane et al. (2025) | Preconception GLP-1 RA useᵃ | Not further reported; pregnancy onset as defined within the network | Federated electronic health record network | 1–2 combined | The weight of the estimate falls in Window 1. Discontinuation timing not separated. TriNetX overlap with Hanif et al. and Banerjee et al. |
| Brown et al. (2026) | GLP-1 RA dispensing within 90 days before the LMP, followed by first-trimester continuation (≥1 further dispensing) or discontinuation | LMP | Claims/dispensing records | 4 | Target trial emulation. Weighted Kaplan–Meier risks were reported, so event counts could not be extracted and the study entered no pool. MarketScan overlap with Cesta et al. |
| Maya et al. (2025) | GLP-1 RA prescription record from 3 years before to 90 days after conception | Conception | Health-system electronic health record prescription entries | 1–3 mixed | The weight of the estimate falls in Window 2; in 66% of exposed pregnancies the last prescription was within six months before conception. Propensity score matching 1:3. Only risk ratios reported for frequent outcomes. |
| Lewin et al. (2026) | GLP-1 RA use at any time during pregnancy; trimester not separated | Not reported; no LMP, conception or onset boundary given | Electronic health record medication data | 3–4 combined | No covariate adjustment was possible because individual-level data were inaccessible. Carries 93.5% of the secondary pregnancy-period malformation analysis weight and 52.6% of the preterm pool weight; preeclampsia not pooled, presented at study level (see Table 1). Counts back-calculated from percentages rounded to one decimal (see Supplementary Table S3). |
| Pondugula et al. (2026) | GLP-1 RA exposure before and in early pregnancy; stratified by treatment indication | Calculated conception date | Electronic medical record query and manual chart abstraction | Variable | Pre-pregnancy stratum assigned to Windows 1–2, within-pregnancy stratum to Window 3, secondary outcomes to the 1–3 mixed window. Gestational weight gain reported according to IOM criteria and therefore not pooled. |
| Banerjee et al. (2026) | Use 6–18 months before the index pregnancy encounter; discontinuation >90 days before (planned) or within ≤90 days (abrupt) | Index pregnancy encounter | Federated electronic health record network | 1 and 2 (separate arms) | Empirical basis for separating Windows 1 and 2. Only a risk ratio was reported for a frequent outcome, so the study entered no pool. TriNetX overlap. |
| Parker et al. (2025) | Unplanned pregnancies arising during clinical development programs; placebo-arm comparator | Not reported; clinical detection of unplanned pregnancy used | Study drug administration records, regulatory files | 3 | Within-source inconsistency: 2 congenital anomalies in a denominator of at least 111 in the aggregate text versus 3 fetal abnormalities in a denominator of 112 in Table 2 of the same publication. Therefore excluded from the malformation pool. |
| Hanif et al. (2025) | GLP-1 RA exposure from 1 year before to 1 month after a first-trimester pregnancy diagnosis | First-trimester pregnancy diagnosis | Federated electronic health record network | 1–3 mixed | A comparator group and propensity score matching were available, but no point estimate or raw count was reported for any outcome; retained in the review, excluded from quantitative synthesis. TriNetX overlap. |

Exposure definitions and time anchors are taken from the primary publications. Assignment followed a deterministic hierarchy: exposure ending >90 days before conception was Window 1; explicitly prepregnancy-only exposure ending 1-89 days before conception was Window 2; day -90 and any exposure thereafter through the first trimester were Window 3; continuation beyond the first trimester was Window 4. If a source-defined interval crossed boundaries and could not be separated, it was retained as a labeled combined window. Thus, Window 2 and Window 3 are mutually exclusive, and Window 3 takes precedence when a source-defined periconceptional interval could include gestational exposure. In studies with multiple comparators, the primary comparator followed the hierarchy in Methods; alternatives were sensitivity analyses. aThe exact preconception interval was not further reported. LMP, last menstrual period; IOM, Institute of Medicine.

**Supplementary Table S2. Nine-scenario sensitivity analyses for rounded event percentages**

Panel A. Major congenital malformation - rounded-percentage sensitivity for the secondary pregnancy-period OR analysis

| **Exposed (%)** | **Comparator (%)** | **Pooled OR (95% CI)** | **p** |
| --- | --- | --- | --- |
| 3.25 | 3.25 | 1.00 (0.94–1.07) | 0.98 |
| 3.25 | 3.30 | 0.98 (0.92–1.05) | 0.65 |
| 3.25 | 3.35 | 0.97 (0.91–1.04) | 0.37 |
| 3.30 | 3.25 | 1.01 (0.95–1.08) | 0.67 |
| 3.30 | 3.30 | 1.00 (0.94–1.07) | 0.99 |
| 3.30 | 3.35 | 0.99 (0.92–1.05) | 0.66 |
| 3.35 | 3.25 | 1.03 (0.96–1.10) | 0.39 |
| 3.35 | 3.30 | 1.01 (0.95–1.08) | 0.68 |
| 3.35 | 3.35 | 1.00 (0.94–1.07) | 0.98 |
| **Range** |  | **0.97–1.02** | **0.23–0.85** |

Because the dominant Lewin cohort reported percentages rounded to one decimal, each true proportion lies within +/-0.05 percentage points of the reported value. Nine lower/reported/upper combinations were evaluated using denominators 35,083 and 108,510. In each scenario the recalculated Lewin OR was combined with the four unchanged Window 3 OR inputs from Cesta, Dao, Kolding and Broe as a secondary pregnancy-period sensitivity analysis. Chou was retained as a source-reported adjusted RR rather than converted to a pooled crude OR because its propensity-score-matched GEE model accounts for the matched design, whereas a simple aggregate 2x2 OR does not. No scenario reached statistical significance. aScenario corresponding to the reported percentages.

***Panel B. Preeclampsia, single cohort (reported percentages, 13.1% vs 11.9%)***

| **Exposed (%)** | **Comparator (%)** | **OR (95% CI)** | **CI includes 1** |
| --- | --- | --- | --- |
| 13.05 | 11.85 | 1.12 (1.08–1.16) | No |
| 13.05 | 11.90 | 1.11 (1.07–1.15) | No |
| 13.05 | 11.95 | 1.11 (1.07–1.15) | No |
| 13.10 | 11.85 | 1.12 (1.08–1.16) | No |
| 13.10ᵃ | 11.90ᵃ | 1.12 (1.08–1.16) | No |
| 13.10 | 11.95 | 1.11 (1.07–1.15) | No |
| 13.15 | 11.85 | 1.13 (1.09–1.17) | No |
| 13.15 | 11.90 | 1.12 (1.08–1.16) | No |
| 13.15 | 11.95 | 1.12 (1.08–1.16) | No |
| **Range** |  | **1.11–1.13** | **No in 9/9** |

Preeclampsia was not pooled because only two independent studies reported it; the values in this panel are unadjusted single-cohort estimates. They show that the nominal association is not attributable to rounding but do not address confounding. ᵃScenario corresponding to the reported percentages.

***Panel C. Urinary congenital malformation, single cohort (reported percentages, 0.5% vs 0.4%)***

| **Exposed (%)** | **Comparator (%)** | **OR (95% CI)** | **CI includes 1** |
| --- | --- | --- | --- |
| 0.45 | 0.35 | 1.29 (1.07–1.55) | No |
| 0.45 | 0.40 | 1.13 (0.94–1.35) | Yes |
| 0.45 | 0.45 | 1.00 (0.84–1.20) | Yes |
| 0.50 | 0.35 | 1.43 (1.20–1.71) | No |
| 0.50ᵃ | 0.40ᵃ | 1.25 (1.05–1.49) | No |
| 0.50 | 0.45 | 1.11 (0.94–1.32) | Yes |
| 0.55 | 0.35 | 1.57 (1.32–1.87) | No |
| 0.55 | 0.40 | 1.38 (1.16–1.63) | No |
| 0.55 | 0.45 | 1.22 (1.04–1.45) | No |
| **Range** |  | **1.00–1.57** | **Yes in 3 of 9** |

Denominators 35,083 and 108,510; each row is an unpooled single-cohort estimate. Because the two reported percentages differ by only 0.1 points, rounding to one decimal moves this estimate far more than those in Panels A and B. ᵃScenario corresponding to the reported percentages and used in the main text. OR, odds ratio; CI, confidence interval.

**Supplementary Table S3. Meta-analysis inputs and weights for major congenital malformation, stillbirth, preterm birth, and small for gestational age (effect-measure-specific primary analyses and legacy sensitivity analyses)**

Panel A. Major congenital malformation - timing-specific Window 3 OR analysis and secondary pregnancy-period sensitivity (one corrective-reanalysis comparator per study)

| Study / analysis | **Primary comparator** | **OR (95% CI)** | **ln(OR)** | **Standard error** | **Weight (%)** |
| --- | --- | --- | --- | --- | --- |
| Cesta et al. (2024) | Insulin (no ADM other than metformin); crude OR | 1.02 (0.79–1.31) | 0.0162 | 0.1312 | 5.92 |
| Dao et al. (2024) | Diabetes reference | 0.98 (0.16–5.82) | −0.0202 | 0.9168 | 0.12 |
| Kolding et al. (2025) | Insulin | 0.64 (0.12–2.15) | −0.4463 | 0.7362 | 0.19 |
| Broe et al. (2025) | Pre-pregnancy discontinuers | 0.74 (0.20–2.70) | −0.3011 | 0.6640 | 0.23 |
| Lewin et al. (2026) | PGDM without GLP-1 RA | 1.00 (0.94–1.07) | 0.0000 | 0.0330 | 93.54 |
| Pooled sensitivity (Window 3 + 3-4) |  | 1.00 (0.94–1.06) |  |  | 100.00 |

**Panel B. Major congenital malformation — exploratory alternative-comparator OR-specific sensitivity**

| **Study** | **Alternative comparator** | **OR (95% CI)** | **ln(OR)** | **Standard error** | **Weight (%)** |
| --- | --- | --- | --- | --- | --- |
| Cesta et al. (2024) | Insulin (crude OR; sole comparator) | 1.02 (0.79–1.31) | 0.0162 | 0.1312 | 35.21 |
| Dao et al. (2024) | Overweight/obese reference | 0.54 (0.11–2.75) | −0.6162 | 0.8212 | 3.21 |
| Kolding et al. (2025)† | Unexposed general population | 1.24 (0.24–4.01) | 0.2151 | 0.7184 | 4.11 |
| Broe et al. (2025)† | Drug-free general population | 3.52 (1.59–7.82) | 1.2585 | 0.4064 | 10.85 |
| Lewin et al. (2026) | PGDM without GLP-1 RA | 1.00 (0.94–1.07) | 0.0000 | 0.0330 | 46.61 |
| Pooled |  | 1.14 (0.64–2.02); I² = 60.8% |  |  | 100.00 |

†These studies used different exposed drug groups and study periods, but their alternative unexposed/drug-free general-population comparator pools may partially overlap within Danish data sources; Panel B is therefore exploratory and not used for primary inference. In the primary analysis the selected comparators were insulin (Kolding et al.) and pre-pregnancy discontinuers (Broe et al.), so the potentially overlapping general-population controls were not used.

**Panel C. Major congenital malformation — legacy rare-outcome approximation sensitivity (comparison-level structure)**

| **Study** | **Comparator** | **Subgroup** | **Effect (95% CI)** | **ln(OR)** | **Standard error** | **Variance handling** | **Weight (%)** |
| --- | --- | --- | --- | --- | --- | --- | --- |
| Cesta et al. (2024) | Insulin (no ADM other than metformin) | Diabetic | aRR 0.95 (0.72–1.26) | −0.0513 | 0.1428 | — | 5.04 |
| Dao et al. (2024) | Diabetes reference | Diabetic | 0.98 (0.16–5.82) | −0.0202 | 1.2966 | ×2ᵃ | 0.06 |
| Kolding et al. (2025) | Insulin | Diabetic | 0.64 (0.12–2.15) | −0.4463 | 1.0411 | ×2ᵃ | 0.09 |
| Chou et al. (2026) | Insulin (other ADMs allowed) | Diabetic | aRR 0.64 (0.11–3.83) | −0.4463 | 0.9057 | — | 0.13 |
| Dao et al. (2024) | Overweight/obese reference | Non-diabetic | 0.54 (0.11–2.75) | −0.6162 | 1.1613 | ×2ᵃ | 0.08 |
| Kolding et al. (2025) | Unexposed general population | Non-diabetic | 1.24 (0.24–4.01) | 0.2151 | 1.0159 | ×2ᵃ | 0.10 |
| Broe et al. (2025) | Drug-free | Non-diabetic | 3.52 (1.59–7.82) | 1.2585 | 0.5747 | ×2ᵃ | 0.31 |
| Broe et al. (2025) | Pre-pregnancy discontinuers | Other | 0.74 (0.20–2.70) | −0.3011 | 0.9390 | ×2ᵃ | 0.12 |
| Lewin et al. (2026) | PGDM without GLP-1 RA | Other | 1.00 (0.94–1.07) | 0.0000 | 0.0330 | — | 94.08 |
| **Pooled** |  |  | 1.00 (0.93–1.08); I² = 0% |  |  |  | **100.00** |

***Panel D. Stillbirth — study-level inputs***

| **Study** | **Primary comparator** | **Events** | **OR (95% CI)** | **ln(OR)** | **Standard error** | **Weight (%)** |
| --- | --- | --- | --- | --- | --- | --- |
| Dao et al. (2024) | Diabetes reference | 1/168 vs 1/156 | 0.93 | −0.0746 | 1.4186 | 21.05 |
| Chou et al. (2026) | Insulin (PS 1:4) | 7/160 vs 13/606 | 2.09 (0.82–5.32) | 0.7357 | 0.4775 | 53.83 |
| Parker et al. (2025) | Placebo | 1/111 vs 2/38 | 0.16 | −1.8101 | 1.2397 | 25.13 |
| **Pooled** |  |  | 0.93 (0.04–22.9) |  |  | 100.00 |

***Panel E. Preterm birth — study-level inputs***

| **Study** | **Primary comparator** | **Events** | **OR (95% CI)** | **ln(OR)** | **Standard error** | **Weight (%)** |
| --- | --- | --- | --- | --- | --- | --- |
| Lewin et al. (2026) | PGDM without GLP-1 RA | 8,104/35,083 vs 22,570/108,510 | 1.14 (1.11–1.18) | 0.1343 | 0.0147 | 52.63 |
| Hviid et al. (2026) | PS-matched controls (liraglutide 1.38; 1.01–1.90 and semaglutide 1.51; 1.06–2.14; within-study combined) | — | 1.44 (1.14–1.82) | 0.3624 | 0.1199 | 38.32 |
| Dao et al. (2024) | Diabetes reference | 9/113 vs 19/126 | 0.49 (0.21–1.13) | −0.7189 | 0.4274 | 9.05 |
| **Pooled** |  |  | **1.16 (0.47–2.82); I² = 73.6%** |  |  | **100.00** |

***Panel F. SGA — study-level inputs***

| **Study** | **Primary comparator** | **Events** | **OR (95% CI)** | **ln(OR)** | **Standard error** | **Weight (%)** |
| --- | --- | --- | --- | --- | --- | --- |
| Hviid et al. (2026) | PS-matched controls (liraglutide 0.93; 0.65–1.34 and semaglutide 1.07; 0.73–1.56; within-study combined) | — | 0.99 (0.77–1.29) | −0.0058 | 0.1336 | 88.14 |
| Dao et al. (2024) | Diabetes reference | 9/113 vs 10/124 | 0.99 (0.39–2.52) | −0.0136 | 0.4791 | 6.85 |
| Chou et al. (2026) | Insulin (PS 1:4) | 4/153 vs 18/593 | 0.86 (0.29–2.57) | −0.1537 | 0.5604 | 5.01 |
| **Pooled** |  |  | 0.99 (0.77–1.26); I² = 0% |  |  | 100.00 |

Standard errors were derived on the log scale from reported 95% CIs or from 2x2 tables for raw-count ORs when compatible with the source design. Primary pools are OR-specific. Cesta et al. (2024) reported an adjusted RR for MCM; its crude OR from published marginal event counts was used in Panels A and B, while the adjusted RR is retained as a study-level estimate. Chou et al. (2026) used 1:4 propensity-score matching, exact matching on maternal age group, infant year and sex, and log-link Poisson GEE. Its propensity score included demographics, lifestyle factors, maternal comorbidities, obstetric comorbidity, health-care utilization, antihypertensive and lipid-modifying therapies, and concomitant antidiabetic medications. The source-reported adjusted RR was retained as the design-consistent MCM estimate; the simple aggregate-count OR was not pooled because it ignores matched-set correlation, not because its direction differed from the adjusted result. Fewer than three adjusted RRs were available on a common scale, so adjusted estimates were not pooled. Panel C preserves the earlier rare-outcome convergence/comparison-level structure only as a legacy sensitivity analysis and is not used for primary inference. In Panels A, B, D, E and F each study contributes a single comparison, so shared exposed-arm splitting and variance inflation are not used. Hviid et al. (2026) agent-specific ORs were combined within study for preterm birth and SGA. The four-study Window 3 MCM analysis gave OR 0.99 (0.77-1.27). Adding Lewin et al. (2026; Window 3-4, 93.5% weight) gave the secondary pregnancy-period estimate OR 1.00 (0.94-1.06). Excluding Lewin from Panel B gave 1.37 (0.42-4.49; I² = 67.7%). In Panel F, excluding Hviid et al. (2026; 88.1% weight) gave OR 0.93 (0.39-2.24).

**Supplementary Table S4. Final consensus Newcastle–Ottawa Scale assessment of the 14 comparative data sources**

***Section A — Selection and Comparability***

| **Study** | **Selection (0–4)** | **Selection rationale** | **Comparability (0–2)** | **Comparability rationale** |
| --- | --- | --- | --- | --- |
| Cesta et al. (2024) | 4 | Population-based registers; comparator from the same source; exposure by dispensing; outcome absent at baseline | 2 | Insulin active comparator; adjustment for birth year, maternal age and obesity |
| Dao et al. (2024) | 3 | Teratology-service attendees are a selected group; comparators from the same services; exposure by structured prospective interview | 2 | Diabetic reference; adjusted estimates |
| Kolding et al. (2025) | 4 | Regional electronic records; same source; medication records; outcome absent at baseline | 0 | Adjustment not possible with n = 32 exposed pregnancies; indication not comparable, as T2DM constituted 25.8% of the insulin group |
| Chou et al. (2026) | 4 | National insurance records; same source; dispensing; outcome absent at baseline | 2 | Insulin comparator; 1:4 propensity score matching |
| Broe et al. (2025) | 4 | National prescription registry; same source; dispensing; outcome absent at baseline | 1 | Pre-pregnancy discontinuers served as the comparator in the primary MCM analysis, partially controlling indication by design; the second star was withheld for insufficient additional adjustment for diabetes, BMI and maternal age |
| Hviid et al. (2026) | 4 | National prescription registry; same source; dispensing; outcome absent at baseline | 2 | Agent-specific propensity score matching; counterfactual BMI scenarios |
| Imbroane et al. (2025) | 4 | Federated EHR network; same network; recorded medication data; outcome absent at baseline | 1 | Propensity score matching; BMI/HbA1c components not reported |
| Brown et al. (2026) | 4 | Claims/dispensing records; same source; dispensing; outcome absent at baseline | 2 | Target trial emulation; weighting |
| Maya et al. (2025) | 4 | Health-system EHR; same source; prescription entries; outcome absent at baseline | 1 | 1:3 propensity score matching; components sparsely reported |
| Lewin et al. (2026) | 3 | Large EHR base; comparator from the same database; the exposure-ascertainment star was withheld because no pregnancy time anchor was reported | 0 | No covariate adjustment possible because individual-level data were inaccessible |
| Pondugula et al. (2026) | 3 | Single center; comparator from the same center; EMR query and manual chart abstraction | 2 | Stratification by indication; adjusted estimates |
| Banerjee et al. (2026) | 4 | Federated EHR network; same network; clearly defined discontinuation timing; outcome absent at baseline | 2 | 1:1 propensity score matching including BMI and HbA1c |
| Parker et al. (2025) | 3 | Trial participants are a selected group; placebo arm from the same programs; exposure precisely ascertained from administration records | 1 | Comparator of randomized origin; balance not guaranteed in the unplanned-pregnancy subset and no adjustment performed |
| Hanif et al. (2025) | 4 | Federated EHR network; same network; recorded medication data; outcome absent at baseline | 1 | Propensity score matching; details not reported |

***Section B — Outcome assessment and overall judgement***

| **Study** | **Outcome (0–3)** | **Outcome rationale** | **Total** | **Overall judgement** |
| --- | --- | --- | --- | --- |
| Cesta et al. (2024) | 2 | Register-linked ascertainment; follow-up to delivery; adequacy star withheld because the analysis was restricted to live births | 8 | Low |
| Dao et al. (2024) | 2 | Prospective ascertainment; follow-up to delivery; adequacy star withheld because major-defect assessment was restricted to live births | 7 | Low |
| Kolding et al. (2025) | 3 | EUROCAT classification; follow-up to delivery; low attrition | 7 | Highᵃ |
| Chou et al. (2026) | 2 | Claims-based ascertainment; follow-up to delivery; internal-consistency star withheld because the MCM estimate could not be reproduced from raw counts | 8 | Low |
| Broe et al. (2025) | 2 | National register ascertainment; follow-up to delivery; adequacy star withheld because more than 700 associations were screened without multiplicity correction | 7 | Low |
| Hviid et al. (2026) | 3 | National register ascertainment; follow-up to delivery; non-reporting of stillbirth counts for confidentiality is a reporting limitation | 9 | Low |
| Imbroane et al. (2025) | 3 | EHR-code ascertainment; complete follow-up | 8 | Low |
| Brown et al. (2026) | 3 | Claims-based ascertainment; follow-up to delivery; low attrition | 9 | Low |
| Maya et al. (2025) | 3 | EHR ascertainment; follow-up to delivery; low attrition | 8 | Low |
| Lewin et al. (2026) | 3 | EHR-code ascertainment; follow-up to delivery; large cohort | 6 | Highᵃ |
| Pondugula et al. (2026) | 3 | Chart-review ascertainment; follow-up to delivery; low attrition | 8 | Low |
| Banerjee et al. (2026) | 3 | EHR-code ascertainment; complete follow-up | 9 | Low |
| Parker et al. (2025) | 2 | Regulatory-file ascertainment; complete follow-up; adequacy star withheld because of a within-source inconsistency in anomaly counts | 6 | Moderate |
| Hanif et al. (2025) | 1 | No point estimate or raw count reported for any outcome | 6 | Highᵇ |

In studies with multiple comparators, the Comparability score refers to the comparator used in the primary meta-analysis; confounding issues affecting alternative comparators were assessed separately in the sensitivity analyses in which those comparators were used. EHR, electronic health record; EMR, electronic medical record; T2DM, type 2 diabetes mellitus.

**Supplementary Table S5. Final consensus Newcastle–Ottawa Scale summary matrix: domain ratings and overall risk-of-bias judgements**

| **Study** | **Selection (0–4)** | **Comparability (0–2)** | **Outcome (0–3)** | **Total** | **Overall judgement** |
| --- | --- | --- | --- | --- | --- |
| Cesta et al. (2024) | ★★★★ | ★★ | ★★☆ | 8 | Low |
| Dao et al. (2024) | ★★★☆ | ★★ | ★★☆ | 7 | Low |
| Kolding et al. (2025) | ★★★★ | ☆☆ | ★★★ | 7 | Highᵃ |
| Chou et al. (2026) | ★★★★ | ★★ | ★★☆ | 8 | Low |
| Broe et al. (2025) | ★★★★ | ★☆ | ★★☆ | 7 | Low |
| Hviid et al. (2026) | ★★★★ | ★★ | ★★★ | 9 | Low |
| Imbroane et al. (2025) | ★★★★ | ★☆ | ★★★ | 8 | Low |
| Brown et al. (2026) | ★★★★ | ★★ | ★★★ | 9 | Low |
| Maya et al. (2025) | ★★★★ | ★☆ | ★★★ | 8 | Low |
| Lewin et al. (2026) | ★★★☆ | ☆☆ | ★★★ | 6 | Highᵃ |
| Pondugula et al. (2026) | ★★★☆ | ★★ | ★★★ | 8 | Low |
| Banerjee et al. (2026) | ★★★★ | ★★ | ★★★ | 9 | Low |
| Parker et al. (2025) | ★★★☆ | ★☆ | ★★☆ | 6 | Moderate |
| Hanif et al. (2025) | ★★★★ | ★☆ | ★☆☆ | 6 | Highᵇ |

Filled stars (★) denote points awarded and open stars (☆) points withheld; domain rationales are given in Supplementary Table S4. Scores shown are the final consensus scores after independent assessment by E.M. and A.N.M. Overall risk-of-bias classification agreed for all 14 studies; three domain-level discrepancies (Kolding: Outcome; Chou: Selection; Pondugula: Selection) were resolved by source re-review and consensus. Overall judgement rule: 7–9 stars low, 5–6 moderate, ≤4 high risk. ᵃRated high irrespective of total score because Comparability = 0. ᵇRated high irrespective of total score because Outcome ≤ 1. In the Selection domain, 10 studies scored 4/4 and 4 scored 3/4; in Comparability, 7 scored 2/2, 5 scored 1/2 and 2 scored 0/2; in Outcome, 8 scored 3/3, 5 scored 2/3 and 1 scored 1/3. The final distribution was 10 low, 1 moderate and 3 high risk, with star loss concentrated in the Comparability domain.

**Supplementary Table S6. GRADE evidence profile for key maternal and fetal outcomes**

| **Outcome** | **Evidence base** | **GRADE domain judgements** | **Certainty** | **Key rationale** |
| --- | --- | --- | --- | --- |
| Major congenital malformation | 4 studies in timing-specific Window 3 OR analysis; 1 trimester-unspecified study added in secondary pregnancy-period analysis; 1 additional adjusted RR retained at study level | Risk of bias: Serious Inconsistency: Not serious Indirectness: Serious Imprecision: Serious Publication bias: Undetected; not assessable quantitatively | Very low | Observational evidence; the primary Window 3 estimate is imprecise (OR 0.99, 0.77-1.27). The secondary pregnancy-period estimate is 93.5% weighted by the high-risk, unadjusted, trimester-unspecified Lewin cohort and therefore cannot provide definitive reassurance. |
| Preterm birth | 3 studies in OR pool | Risk of bias: Serious Inconsistency: Serious Indirectness: Serious Imprecision: Serious Publication bias: Undetected; not assessable quantitatively | Very low | Lewin is unadjusted and contributes 52.6% of weight; I²=73.6%; exposure timing includes Window 3–4; CI 0.47–2.82 permits clinically important benefit or harm. |
| Small for gestational age | 3 studies in OR pool | Risk of bias: Not serious Inconsistency: Not serious Indirectness: Not serious Imprecision: Serious Publication bias: Undetected; not assessable quantitatively | Very low | Only three studies; one study contributes 88.1% of weight. Dominant-study exclusion gives OR 0.93 (0.39–2.24), leaving substantial imprecision. |
| Stillbirth | 3 studies in OR pool | Risk of bias: Serious Inconsistency: Not serious Indirectness: Not serious Imprecision: Very serious Publication bias: Undetected; not assessable quantitatively | Very low | Very sparse events and extremely wide pooled CI (0.04–22.9); one source is a selected regulatory-development pregnancy subset and another contributes only crude counts. |
| Preeclampsia | 2 studies; not pooled | Risk of bias: Serious Inconsistency: Not assessable Indirectness: Serious Imprecision: Serious Publication bias: Undetected; not assessable quantitatively | Very low | Only two independent studies; the nominal association is from an unadjusted trimester-unspecified cohort, whereas the propensity-score-matched study is compatible with no association. |

GRADE certainty starts at low for observational evidence and was downgraded when serious limitations were present. Because certainty cannot be reduced below very low, multiple serious limitations may converge on the same final rating. Publication bias could not be assessed because no outcome had at least 10 independent studies. MCM certainty is very low because the timing-specific Window 3 estimate is imprecise and the apparently precise secondary pregnancy-period estimate is dominated by a high-risk, unadjusted, trimester-unspecified cohort.

**Supplementary Table S7. Study-level outcome definitions, ascertainment, and pregnancy-outcome coverage**

| **Study** | **Outcome definition / ascertainment** | **Live-birth restriction** | **Pregnancy losses / terminations** |
| --- | --- | --- | --- |
| Cesta et al. (2024) | Major congenital malformations identified from national registers/claims using source algorithms. | Yes; malformation analysis restricted to live births. | Pregnancy losses and terminations were not included in the MCM analysis. |
| Dao et al. (2024) | Major birth defects assessed prospectively by teratology information services; other pregnancy outcomes recorded during follow-up. | Major-defect analysis restricted to live births. | Spontaneous loss and elective termination were reported separately. |
| Kolding et al. (2025) | Major malformations classified according to EUROCAT from regional electronic records. | Malformation outcome evaluated among live-born infants. | Loss/termination capture was not sufficiently reported for comparative malformation analysis. |
| Chou et al. (2026) | MCM recorded by the attending clinician within 7 days of delivery using birth-certificate/ICD-10 codes; stillbirth >=20 weeks. | MCM, preterm birth, and SGA analyses were birth-record based; preterm/SGA restricted to live births. | Losses before 20 weeks were not captured; chromosomal anomalies and known teratogen exposure were excluded. |
| Broe et al. (2025) | Congenital outcomes identified in Danish registry screening using prespecified drug-outcome algorithms. | Comparative congenital-malformation estimate was birth-registry based. | Separate loss/termination ascertainment was not extractable for the MCM estimate. |
| Hviid et al. (2026) | Registry-based preterm birth, SGA, LGA, preeclampsia, and related outcomes; agent-level estimates reported for liraglutide and semaglutide. | Birth outcomes were evaluated among eligible births. | Stillbirth counts were suppressed for confidentiality; early-loss/termination data were not available for pooling. |
| Imbroane et al. (2025) | Hypertensive disorders identified from federated EHR diagnosis data. | Not applicable to a congenital-malformation analysis. | Not reported as study outcomes. |
| Brown et al. (2026) | Target-trial-emulation outcomes included non-live birth and birth-weight categories using claims/dispensing data. | Live-birth outcomes analyzed separately from non-live birth. | Non-live birth was captured as a composite; component-specific counts were not extractable. |
| Maya et al. (2025) | Maternal gestational weight gain and pregnancy outcomes obtained from health-system EHR data. | Congenital malformation was not a principal extractable outcome. | Loss/termination definitions were not available for synthesis. |
| Lewin et al. (2026) | Congenital-malformation categories and obstetric outcomes identified from Epic Cosmos EHR codes; source percentages were rounded. | Source did not clearly report a live-birth-only restriction for every malformation category. | Separate capture of early losses and terminations was not reported. |
| Pondugula et al. (2026) | HDP and gestational weight gain obtained by EHR query with manual chart abstraction; GWG categorized by IOM criteria. | Not applicable to MCM. | Not principal study outcomes. |
| Banerjee et al. (2026) | Maternal outcomes identified from federated EHR data in planned- versus abrupt-discontinuation cohorts. | Not applicable to MCM. | Not reported as extractable comparative outcomes. |
| Parker et al. (2025) | Pregnancy and fetal outcomes abstracted from clinical-development/regulatory records; congenital/fetal abnormality definitions were inconsistent across source sections. | Not consistently specified. | Pregnancy losses and terminations were reported, but outcome denominators/labels were not sufficiently consistent for MCM pooling. |
| Hanif et al. (2025) | Outcome concepts were defined in the federated EHR analysis, but no numerical point estimate or event count was reported. | Not determinable from extractable data. | Not numerically reported. |

MCM, major congenital malformation; SGA, small for gestational age; LGA, large for gestational age; EHR, electronic health record; EUROCAT, European network of population-based registries for the epidemiological surveillance of congenital anomalies; IOM, Institute of Medicine; NR, not reported.

**Supplementary Table S8. Agent-specific exposure and outcome reporting**

| **Study** | **Agents represented** | **Agent-specific exposed N** | **Agent-specific comparative outcome available?** |
| --- | --- | --- | --- |
| Cesta et al. (2024) | Multiple GLP-1 RAs | Not extractable by agent for pooled outcomes | No; class-level estimate |
| Dao et al. (2024) | Liraglutide, semaglutide, dulaglutide, exenatide | Reported for the exposed cohort, but outcome-specific denominators/events were not separable | No agent-specific comparative estimate suitable for pooling |
| Kolding et al. (2025) | Semaglutide | 32 | Yes, study-level only; too sparse for agent-specific meta-analysis |
| Chou et al. (2026) | Multiple GLP-1 RAs | 160 class-level | No |
| Broe et al. (2025) | Multiple individual agents in drug-level screening | Outcome-specific agent counts sparse/not consistently extractable | No meta-analyzable agent-specific MCM estimate |
| Hviid et al. (2026) | Liraglutide; semaglutide | 295; 234 | Yes for selected outcomes; combined within study where required |
| Imbroane et al. (2025) | Class-level GLP-1 RA | 4,267 class-level | No |
| Brown et al. (2026) | Multiple GLP-1-based therapies | 3,572 class-level | No extractable comparative result by individual agent |
| Maya et al. (2025) | Class-level GLP-1 RA | 448 class-level | No |
| Lewin et al. (2026) | Class-level GLP-1 RA | 35,083 class-level | No |
| Pondugula et al. (2026) | Multiple GLP-1 RAs | 243 class-level | No |
| Banerjee et al. (2026) | Class-level GLP-1 RA | 735 class-level | No |
| Parker et al. (2025) | Clinical-development agents including GLP-1 RAs/dual agonist programs | >=111 aggregate pregnancies | Agent-level denominators/outcomes not sufficiently consistent for pooling |
| Hanif et al. (2025) | Class-level GLP-1 RA | NR | No numerical estimate |

Agent-specific meta-analysis was not performed because fewer than three independent studies reported comparable outcomes for the same agent, window, comparator, and effect measure. No extractable comparative tirzepatide-specific pregnancy outcome was available. Class-level findings must not be interpreted as evidence of safety for any individual agent.
